# Supplementary material for: FAPI PET versus FDG PET, CT or MRI for Staging Pancreatic-, Gastric- and Cholangiocarcinoma: Systematic Review and Head-to-Head Comparisons of Diagnostic Performances
Source: Diagnostics (Basel). 2022 Aug 12;12(8):1958. doi: 10.3390/diagnostics12081958 (PMC9406684; doi:10.3390/diagnostics12081958)
Supplement: Supplementary file 1 [file diagnostics-12-01958-s001.zip › diagnostics-1803282-supplementary.pdf]

Table S1. Sens analysis; as reported in the original manuscript or calculated based on published raw data.

YEAR	AUTHOR	TYPE OF CANCER	**PATIENT-WISE ANALYSIS** sensitivity of FAPI PET for T/N/M/PC (confirmed by reference standard)	**PATIENT-WISE ANALYSIS** sensitivity of FDG PET or CECT/MRI for T/N/M/PC (confirmed by reference standard)	**LESION-WISE ANALYSIS** sensitivity of FAPI PET for T/N/M/PC (confirmed by reference standard)	**LESION-WISE ANALYSIS** sensitivity of FDG PET/CT/MRI for T/N/M/PC (confirmed by reference standard)
2021	Chen, H., et al. [10]	P	-	-	-	-
		G	-	-	-	-
		Ch	-	-	-	-
2021	Guo, W., et al. [11]	Ch	100% (7/7) / - / - / -	PET 57% (4/7) / - / - / -<br>CECT 100% (7/7) / - / - / -<br>MRI 100% (7/7) / - / - / -	- / - / 85% (41/48) / -	PET - / - / 52% (25/48) / -<br>CECT not performed<br>MRI - / - / 100% (48/48) / -
2021	Pang, Y., et al. [12]	G	100% (11/11) / - / - / -	36% (4/11) / - / - / -	n.p.	n.p.
2021	Zhao, L., et al. [17]	P<br>G	- / - / - / 100% (6/6)<br>- / - / - / 100% (13/13)	- / - / - / 67% (4/6)<br>- / - / - / 54% (7/13)	-	-
2021	Qin, C., et al. [13]	G	100% (14/14) / - / - / -	71% (10/14) / - / - / -	n.p.	n.p.
2021	Rohrich, M., et al. [14]	P	-	PET n.p.<br>CECT -	*n.p.*	*n.p.*
2021	Shi, X., et al. [15]	Ch	100% (3/3) / - / - / -	100% (3/3) / - / - / -	100% (4/4) / - / - / -	100% (4/4) / - / - / -
2021	Shi, X., et al. [16]	G<br>Ch	- / - / - / -<br>100% (2/2) / - / - / -	PET n.p.<br>CECT -<br>MRI -	-	PET n.p.<br>CECT -<br>MRI -
2021	Pang, Y., et al. [18]	P	100% (26/26) / - / - / -	PET 73% (19/26) / - / - / -<br>CECT - / - / - / -	- / 82% / bone 92%, visceral 88% / -	PET - / 59% / bone 44%, visceral 48% / -<br>CECT - / - / - / -

2021	Kuten, J., et al. [19]	G	100% (10/10) / 100% (2/2) / - / 100% (5/5)	50% (5/10) / 100% (2/2) / - / 0% (0/5)	-	-

- not reported for the diseases of interest separately

n.p. analyses not performed in the study

Table S2. SUVmax and TBRmax values for FAPI and FDG

Year	Author	TYPE OF CANCER	PATIENT-WISE ANALYSIS median SUVmax for T/N/M/PC (nr. of patients), and (TBR) for FAPI	PATIENT-WISE ANALYSIS median SUVmax for T/N/M/PC (nr. of patients), and (TBR) for FDG	LESION-WISE ANALYSIS median SUVmax for T/N/M/PC (nr. of patients), and (TBR) for FAPI	LESION-WISE ANALYSIS median SUVmax for T/N/M/PC (nr. of patients), and (TBR) for FDG
2021	Chen, H. [10]	P	24.8 (1) / - / - / - (- / - / - / -)	2.7 (1) / - / - / - (- / - / - / -)	-	-
		G	14.1 (5) / - / - / - (- / - / - / -)	2.7 (5) / - / - / - (- / - / - / -)	-	-
		Ch	10.3 (1) / - / - / - (- / - / - / -)	1.8 (1) / - / - / - (- / - / - / -)	-	-
2021	Guo, W. [11]	Ch	16.5 (7) / - / - / - (7.0/ - / - / -)	4.2 (7) / - / - / - (1.5 / - / - / -)	n.p.	n.p.
2021	Pang, Y. [12]	G	12.7 (11) / - / - / - (- / - / - / -)	3.7 (4) / - / - / - (- / - / - / -)	n.p.	n.p.
2021	Zhao, L. [17]	P<br>G	- / - / - / 7.8 (6) (- / - / - / -)<br>- / - / - / 8.1 (13) (- / - / - / -)	- / - / - / 3.3 (6) (- / - / - / -)<br>- / - / - / 3.4 (13) (- / - / - / -)	-	-
2021	Qin, C. [13]	G	11.3 (14) / 9.9* (12) / - / 8.4 (10) (12.4 / 10.9 / - / 9.6)	6.2 (10) / 6.6 (10) / - / 7.6 (4) (4.0 / 4.0 / - / 5.0)	- / 8.7* (45) / - / 7.6 (42) (- / 9.4 / - / 8.7)	- / 6.1 (33) / - / 5.7 (14) (- / 3.7 / - / 3.7)
2021	Rohrich, M. [14]	P	13.4 (19) / 14.1 (19) / 7.3 (19) / -	*n.p.*	*n.p.*	*n.p.*
2021	Shi, X. [15]	Ch	14.4 (4) / - / - / - (26.5 / - / - / -)	9.2 (4) / - / - / - (4.4 / - / - / -)	- / - / 8.0 (17) / - (- / - / 15.2 / -)	- / - / 4.9 (17) / - (- / - / 2.1 / -)
2021	Shi, X. [16]	G<br>Ch	- / - / - / - (- / - / - / -)	*n.p.*	- / - / - / - (- / - / - / -)<br>- / - / - / -	*n.p.*

			13.6 (2) / - / - / - (26.9 / - / - / -)		(- / - / - / -)	
2021	Pang, Y. [18]	P	21.4 (26) / - / - / - (- / - / - / -)	4.8 (19) / - / - / - (- / - / -/ -)	21.4 (26) / 8.6 (45) / - # / 8.4 (77)	4.8 (19) / 2.7 (23) / -# / 2.8 (33)
**2021**	Kuten, J.	G	15.9 (10) / 4.3 (2) / - / - (3.2 / - / - / -)	5.5 / 7.9 (2)/ - / - (11.9 / - / - / -)	-	-

- not reported for the diseases of interest separately

n.p. analyses not performed in the study

** Patient-wise SUVmax of 9.9 versus lesion-wise SUVmax of 8.7 may concern a typo in the original manuscript.*
